# Antigen-directed p53 restoration in *TP53*-mutated myeloid neoplasms: a hypothesis and theory perspective

**DOI:** 10.3389/fonc.2026.1874395

**Published:** 2026-08-11

**Authors:** Georgio Medawar, Sadie Pyle, Praneeth Baratam, Alexander Coltoff

**Affiliations:** 1 Department of Hematology-Oncology, Medical University of South Carolina, Charleston, SC, United States; 2 College of Pharmacy and Pharmaceutical Sciences, University of Toledo, Toledo, OH, United States

**Keywords:** acute myeloid leukemia, myelodysplastic syndrome, *TP53* mutation, adenovirus, lipid nanoparticles, mRNA, p53 restoration

## Abstract

*TP53*-mutated myeloid neoplasms, including acute myeloid leukemia (AML) and myelodysplastic syndromes (MDS), remain among the hardest-to-treat hematologic malignancies, with short survival despite hypomethylating agents +/- venetoclax, and allogeneic transplantation. Despite encouraging early-phase data, Phase 3 trials of mutant-p53 reactivators to date have not yet translated into clear, practice-changing survival benefits, highlighting a need to explore complementary strategies that aim to directly restore wild-type p53 function in the leukemic niche. Building on clinical experience with the adenoviral p53 product (rAd-p53) in solid tumors, where intratumoral rAd-p53 achieves high cumulative response rates with manageable toxicity, we investigate the feasibility of translating p53 replacement into select *TP53*-mutated myeloid neoplasms using antigen-directed delivery systems. Advances in AML surface proteomics and immunotherapy identify many antigens of interest, in particular, CD33, CD123, and CD209, as internalizing myeloid antigens that are broadly expressed on leukemic blasts and stem/progenitor cells. In parallel, emerging data on *in vivo* gene delivery via lipid nanoparticles (LNP) and adenovirus platforms that are capable of efficient marrow transduction demonstrate that systemic gene transfer to hematopoietic compartments is feasible. We outline a framework in which CD33-, CD123-, and CD209-directed mRNA/LNP systems or leukemia-adapted rAd-p53 vectors could potentially be used to restore wild-type *TP53* in *TP53*-mutant myeloid clones and discuss key preclinical questions in xenograft models. We acknowledge that the strategy is built on converging but largely indirect lines of evidence rather than direct experimental data in *TP53*-mutated AML/MDS, and we explicitly discuss key preclinical validation requirements and biological barriers, including dominant-negative stoichiometry, liver sequestration, and by conceivable toxicities, that must be systematically addressed before clinical translation.

## Introduction

*TP53*-mutated myeloid neoplasms, encompassing acute myeloid leukemia (AML) and myelodysplastic syndromes (MDS), represent the most difficult-to-treat heterogeneous group of diseases in contemporary hematology. *TP53* mutations are highly heterogeneous and depend on mutation type, allelic state, variant allele fraction (VAF), and co-occurring cytogenetic abnormalities such as del(17p) or copy-neutral loss of heterozygosity (CN-LOH). Missense mutations in the DNA-binding domain, which account for >70% of *TP53* alterations, may impair p53 transcriptional activity to varying degrees; hotspot dominant-negative mutants can suppress wild-type p53 function, while biallelic or high-VAF disease reflects near-complete loss of p53 activity. These varied molecular contexts are associated—to differing degrees—with genomic instability, immune evasion, and an inflammatory, apoptosis-resistant marrow microenvironment, rendering conventional cytotoxic agents largely ineffective (1, 2). Often associated with complex karyotypes and rapid clonal evolution, these malignancies confer a dismal prognosis, with median overall survival often measured in months (1). Current therapeutic strategies, including hypomethylating agents (HMAs) +/- venetoclax, and allogeneic hematopoietic cell transplantation, frequently fail to offer durable responses (3). While novel small molecules attempting to reactivate mutant p53 have biological plausibility and *in vitro* activity, they have yet to deliver transformative survival benefits in randomized trials (4, 5). In particular, APR-246 (eprenetapopt), which covalently modifies cysteine residues in mutant p53, in combination with azacitidine demonstrated a remission (CR) rate of 33% and an overall response rate (ORR) of 69%, with an overall survival (OS) of 11.8 months in a Phase 2 trial but failed to demonstrate significant CR and OS benefit compared to azacitidine monotherapy in a Phase 3 trial (6, 7). Rezatapopt (PC14586), which binds with high affinity to the thermolabile pocket created by the *TP53*-Y220C mutation, stabilizes the mutant p53 protein in a wild-type-like conformation and restores p53 transcriptional activity; it does not chemically convert the mutant residue to wild-type, is selective for the Y220C subset, and has shown limited/no apoptotic activity in *TP53*-Y220C AML models (8). Its use in combination with azacitidine is currently being evaluated in a Phase Ib trial (8). These data underscore the need for exploring an alternative approach to restore p53 function. This Hypothesis and Theory manuscript proposes leveraging antigen-directed delivery of either mRNA or adenoviral vectors to deliver and reconstitute wild-type (WT) p53 function within the leukemic marrow niche. We acknowledge that our proposal is built on converging yet largely indirect lines of evidence, and we explicitly frame it as a plausible but unvalidated conceptual framework that requires rigorous preclinical testing.

## Rationale for p53 replacement and the experience in solid tumors

Functional p53 restoration via gene therapy has been evaluated preclinically and clinically across various solid tumor types. Gene therapy functions by substituting faulty genes with normal ones, and therapeutic agents are typically delivered using viral vectors or genetically modified microorganisms (9). *Gendicine^®^*, developed by Shenzhen SiBiono GeneTech, is a replication-defective recombinant adenovirus in which the E1 region is replaced by a Rous sarcoma virus promoter linked with the human wild-type p53 gene and a poly (A) tail (rAd-p53) (10, 11). It was SFDA-approved in China in 2003 and has been used since then, primarily in head and neck squamous cell carcinoma, where intratumoral administration leads to local overexpression of p53, enhanced apoptosis, and sensitization to chemotherapy and radiotherapy, with over 90% cumulative response rate (11). rAd-p53 results in sustained expression of wild-type p53 *in vivo* with manageable toxicity, characterized mainly by transient flu-like symptoms and injection site reactions. To our knowledge, the company made no attempt to expand its use beyond China, making the data difficult to generalize or apply universally. In parallel, INGN-201 (*Advexin^®^*), developed by Introgen Therapeutics, received orphan drug designation from the US FDA based on promising phase 1/2 results in head and neck and non-small cell lung cancer. However, in 2008, the FDA assessed that the Biologics License Application (BLA) for Advexin was not sufficiently complete for review (10). There is currently no FDA-approved p53 gene therapy in the US, but multiple pre-clinical and early-phase clinical trials are evaluating its use in combination with immune checkpoint inhibition in solid tumors (12, 13).

## Preclinical data on the use of rAd-p53 in multiple myeloma

Wang et al. showed that overexpression of WT-p53 inhibits MM tumor cell survival and proliferation both *in vivo* and *in vitro*, and when combined with bortezomib, significantly improves treatment efficacy (14). The group used small interfering RNA (siRNA) with a specific sequence (GGGTTAGTTTACAATCAGC) to knockdown endogenous p53 expression of MM1S cells and transfected them with rAd-p53 using Lipofectamine^®^ 3000. The proliferation of MM1S cells was inhibited by rAd-p53 treatment in a concentration-dependent manner (with an optimal concentration of 5 x 10^11^ VP/ml) by promoting p21 expression and reducing cell cycle protein B1 expression of MM1S. This was validated *in vivo* models and by combining rAd-p53 with bortezomib, a synergistic anti-tumor effect was observed (14).

## Challenges in *TP53*-directed gene therapy in myeloid malignancies

The experience in solid tumors and the pre-clinical data of rAd-p53 use in MM provides a conceptual template for *TP53* gene replacement in the bone marrow niche in patients with myeloid malignancies. Restoring wild-type p53 activity offers the possibility of re-engaging canonical p53 programs, including cell-cycle arrest, apoptosis, senescence, and DNA-damage responses (1, 4, 15). This could theoretically sensitize the leukemic cells to chemotherapy (e.g., HMA) or other targeted therapy. However, several challenges need to be addressed.

First, selecting the appropriate group of *TP53* mutations from a highly heterogeneous pool is needed. Although more than 70% of *TP53* mutations are missense clustering within the DNA-binding domain, various genetic aberrations in *TP53* and chromosomal abnormalities (e.g., del17p, CN-LOH) result in complex and varied functional consequences in MDS and AML (16). *P53*-null and hotspot *TP53* mutations behave more aggressively than non-hotspot mutations activity. Residual transcriptional activity in non-hotspot mutants is mutation-specific and context-dependent and cannot be uniformly generalized; while some retain partial function, others may be functionally null (2, 16). Furthermore, multihit disease status (e.g, high variant-allele-fraction VAF, presence of ≥ 2 mutations), whose definition differs between ICC and WHO-5, confers a significantly worse prognosis compared to single hit (1, 2). At the same time, multihit *TP53* and/or hotspot *TP53* mutations might be the best candidates for p-53 gene therapy due to their dominant impact on disease biology.

A critical consideration for WT-*TP53* restoration in missense-mutant disease is the stoichiometric relationship between delivered WT-p53 and endogenous mutant p53. p53 functions as a tetramer, and dominant-negative mutants such as p53-R248Q have a longer protein half-life than WT-p53, yielding an approximate ratio of 3–4 mutant molecules per WT molecule under endogenous conditions (17). If the vector-delivered WT-p53 is present at a lower stoichiometric level than the endogenous mutant pool, newly synthesized WT-p53 may preferentially form mixed heterotetramers with mutant p53, losing transcriptional activity. Therefore, achieving supraphysiological transgene expression levels—sufficient to shift the WT: mutant ratio toward predominantly WT-p53 homotetramers—will be critical. Potential strategies include: (i) myeloid-specific promoters (e.g., the CD33 promoter or PU.1/SPI1-driven regulatory elements) to achieve high-level transgene expression selectively in malignant myeloid cells (18); (ii) RNA gene-writing tools capable of correcting the *TP53* point mutation at the DNA level, thereby eliminating the source of dominant-negative protein; and (iii) combinatorial approaches pairing W*T-p53* delivery with selective degradation of mutant p53, for example, using targeted protein degraders (17).

Second, the absence of an immunophenotypic signature for *TP53*-mutated myeloid neoplasms makes it difficult to selectively target *TP53*-mutated clones. While Guarnera et al. found that CD38 aberrancy was associated with *TP53* mutation in patients with MDS, another study by Park et al. showed no notable differences in the expression of cell lineage markers, including B-cell, T-cell, and NK-cell markers, among *TP53*-mutated and non-TP53-mutated AML patients (19, 20). Thus, a potential concern would be prolonged bone marrow toxicity due to off-target effects on non-leukemic progenitor stem cells, similar to what was seen in CAR T cell trials with myeloid antigen targeting (CD33, CD123, and CLEC12A) (20). For those reasons, *TP53* gene therapy could be potentially most useful clinically in the pre-transplant setting or part of the conditioning regimen, where myeloablation is desired. Targeting myeloid markers and strategies to modulate target antigen expression will be discussed in the next section.

Third, the delivery system of the *TP53* gene therapy to the bone marrow niche warrants investigation. In the case of solid tumors, the therapy is administered locally (intratumoral or intra-arterial), and the consensus is not to administer intravenously (IV) since rAd-p53 on its own lacks specificity to the tumor cells and may lead to serious complications, such as liver failure or pulmonary damage, which might be caused by the aggregation of rAd-p53 in the lung and liver when given systemically (9). Though limited to a few case reports, intramedullary leukemia-directed therapy has been reported in AML, and can be considered as a route for gene therapy delivery (21, 22). However, systemic IV therapy is less invasive and more practical, and we believe the focus should be on developing methods to deliver WT-p53 to the marrow niche with maximal specificity and minimal off-target systemic side effects.

A particularly important barrier to systemic IV delivery is rapid hepatic sequestration. Unmodified rAd-p53 and standard LNPs are preferentially cleared by Kupffer cells and hepatic sinusoidal endothelium through the reticuloendothelial system (9, 23). Surface-conjugated targeting ligands (e.g., anti-CD209) increase target specificity but do not fully abrogate liver uptake. Approaches that may mitigate this problem include: (i) incorporation of miR-122 binding sites into the 3’ UTR of the mRNA payload – exploiting the liver-specific expression of miR-122 to silence transgene expression in hepatocytes while preserving expression in target myeloid cells, a strategy validated in cardiac and hematologic gene therapy contexts (24, 25); (ii) stealth polymer shielding (e.g., PEGylation or polysarcosine coating) to reduce opsonization and Kupffer cell recognition; and (iii) temporary pharmacological macrophage blockade prior to vector administration, though this approach carries immunosuppressive risks. Co-use of miR-122 detargeting with antigen-directed ligands represents a potentially synergistic dual-layer specificity strategy.

## Candidate myeloid surface targets for WT-*TP53* delivery

The antigen-directed targeted therapies, anti-CD33 and anti-CD123, are currently used in the treatment of specific types of AML and blastic plasmacytoid dendritic cell neoplasm (BPDCN), respectively (26–28). Targeting CD33 and CD123 in AML is challenging due to pronounced antigen heterogeneity across blasts and leukemic stem cells and shared expression on normal HSPCs, resulting in on-target/off-tumor toxicity (29). CD33 and CD123, as well as many other antigens of interest (e.g., CD47, CD70, FLT3, U5 snRNP200, and CLL-1) are being studied in targeted and cellular therapies (30). Strategies are being explored to improve their safety, such as therapeutic index increase (e.g., next generation CD123 antidrug conjugate (ADC)), pharmacologic modulation (e.g., HMAs to upregulate CD123), and genetic epitope engineering (e.g., CRISPR/Cas9 to knockout IL3RA or CD33) (28, 31–33). Epitope editing of donor HSPCs—first reported for CD123 (Marone et al., J Exp Med 2023 (34); Casirati et al., Nature 2023 (35), FLT3 and KIT (Casirati et al., Nature 2023 (35)), and CD33 (Lehnertz et al., ASH 2024 (36))—enables these cells to resist targeted immunotherapy while retaining hematopoietic function, illustrating that antigen-directed strategies can be designed with safety profiles that spare normal progenitors. These antigens and strategies can be theoretically extrapolated to *TP53* gene therapy. Here, we focus on CD33, CD123 and CD209 as target antigen candidates. The selection of these receptors as priority delivery targets was guided by: (i) broad expression on AML blasts and leukemic stem/progenitor cells; (ii) established receptor-mediated internalization capacity; (iii) availability of validated targeting reagents; and (iv) differential expression relative to normal HSPCs. We acknowledge that expression specifically on *TP53*-mutated AML/MDS, as opposed to AML broadly, has not yet been systematically characterized for any of these antigens, and that other myeloid targets (e.g., CD47, FLT3, CLL-1, TIM-3) may also merit consideration. Future surfaceome studies in *TP53*-mutated primary specimens will be needed to refine target prioritization.

### CD33

CD33 is expressed on 88% of AML blasts and on leukemic stem cell–enriched populations and has been clinically validated as a payload receptor by gemtuzumab ozogamicin in patients with favorable-risk AML (26, 29) Recent pre-clinical data showed that CD33-targeted nanoparticles carrying an epigenetic modulator internalized efficiently into CD33-positive AML1-ETO rearranged AML cells, inhibited oncogenic programs, and reduced leukemic burden in patient-derived xenograft (PDX) models (37). These data suggest that CD33-decorated nanoparticles or antibody scaffolds could deliver WT-*TP53* constructs into leukemic blasts and stem/progenitor cells (37).

### CD123

CD123 is overexpressed on high-risk MDS blasts and leukemic stem cells, with a more restricted pattern on normal progenitors, and has been explored as a therapeutic target (38). Recent studies show promising preclinical and early clinical activity across CD123-directed antibodies, ADCs, and cellular therapies, especially in combination regimens, reflecting a potential therapeutic target for AML (39, 40). A dual-targeting CD33/CD123 nanobody T-cell engager eradicated AML cells and leukemic stem cells *in vitro* and in xenograft models while maintaining a favorable safety profile, demonstrating that simultaneous interrogation of both antigens was feasible (41). The same dual-antigen logic could be used to gate uptake of WT-*TP53*-carrying nanoparticles or viral vectors into double-positive leukemic cells and increase specificity.

### CD209 (DC-sign)

Unbiased, high-resolution surfaceome profiling of primary AML specimens has identified CD209 as a canonical myeloid surface antigen that is markedly upregulated on AML blasts but absent on normal HSCs (42). Barpanda et al. showed that CD209 emerged among approximately 150 significantly upregulated surface proteins in newly diagnosed and relapsed AML compared with remission samples. Expression was validated by flow cytometry in multiple AML cell lines and primary samples. A CD209-targeting MMAE-based ADC displayed potent antigen-dependent cytotoxicity (IC50 in the low-nanomolar range) and prolonged survival in a disseminated AML xenograft model, confirming both specificity and internalization capacity. Importantly, CD209 was not detected on normal HSCs in that dataset, making it an attractive candidate (42). An important caveat is that CD209 is physiologically expressed on mature dendritic cells (DCs) and certain tissue macrophages, where it mediates pathogen recognition and antigen presentation (43). Delivering WT-p53 to CD209+ mature DCs or macrophages could theoretically induce p53-dependent apoptosis in these cells, impairing innate immunity and potentially resulting in secondary immunodeficiency or reduced antitumor immunity. To mitigate this risk, several approaches may be considered: (i) restricting WT-*TP53* transgene expression to the malignant myeloid lineage through leukemia-specific transcriptional regulatory elements, such as the CD33 promoter (which contains a PU.1/SPI1-binding element critical for myeloid-restricted expression [48]) or other SPI1/PU.1-driven enhancers, thereby limiting p53 expression to CD33+/SPI1high malignant blasts rather than CD209+ mature immune cells; (ii) combinatorial antigen gating, requiring co-expression of CD209 with CD33 or CD123 for vector internalization, which would exclude CD209-single-positive DCs; and (iii) careful monitoring of DC and macrophage counts and function in preclinical humanized models to assess the clinical relevance of this risk.

## Vehicles for p53 restoration: mRNA/LNP platforms and adenoviral vectors

Two complementary delivery platforms appear particularly relevant for WT-*TP53* restoration in *TP53*-mutated myeloid diseases: non-integrating RNA/LNP systems and tropism-modified adenoviral vectors.

## mRNA and RNA gene-editing platforms

Lipid nanoparticle (LNP) technology is maturing, and systemic, *in vivo* delivery to hematopoietic stem cells (HSC) and progenitor cells could be feasible. Targeted LNPs have been shown to deliver mRNA efficiently to HSC in preclinical models, achieving robust protein expression without *ex vivo* manipulation (44, 45). Tozzi et al. demonstrated that “RNA Gene Writers” delivered via proprietary HSC-targeting LNPs can introduce precise edits into long-term HSCs *in vivo*, including correction of the sickle cell–causing HBB E6V mutation. In humanized NBSGW mice and non-human primates, a single intravenous dose of these LNPs resulted in >90% delivery of a reporter payload to long-term HSCs and >60–70% gene rewriting (i.e., B2M knockout) in stem cells, with sustained engraftment and without evidence of DNA-damage responses associated with nuclease-based editing (46). It is important to note that these HSC-tropic LNP results were achieved using ligands that target normal HSPCs, not leukemic blasts. Substituting HSC-tropic targeting moieties with anti-CD33, anti-CD123, or anti-CD209 ligands is not a simple modular substitution: it will require re-engineering of lipid composition, ligand density, and surface chemistry to optimize uptake specifically by leukemic stem/progenitor cells and will need to be validated in AML-specific primary models. Direct evidence that LNP/mRNA platforms can selectively transduce *TP53*-mutated AML blasts or leukemic stem cells, as opposed to normal marrow compartment, has not yet been established and represents a key gap requiring preclinical investigation.

These findings nonetheless establish a critical proof of principle: LNP-based RNA systems can modify bone marrow stem/progenitor gene expression after systemic administration. The same framework could be repurposed for *TP53*-mutated myeloid neoplasms by: 1) Switching from HSC-tropic targeting ligands to anti-CD33, anti-CD123, or anti-CD209 antibodies or ligands on the LNP surface, 2) Replacing the gene-editing payload with an optimized WT-*TP53* mRNA construct (for p53 replacement) or an RNA gene-writing cassette that corrects *TP53* mutations at the DNA level in leukemic stem and progenitor cells.

Early clinical experience with *in vivo* gene delivery further strengthens this rationale. In the inMMyCAR phase 1 study (KLN-1010), fusogen-engineered lentiviral particles administered intravenously to patients with relapsed/refractory multiple myeloma selectively transduced circulating T cells *in vivo* to generate anti-BCMA CAR-T cells, achieving MRD-negative responses without apheresis or lymphodepletion (47). These results indicate that *in vivo* gene transfer to immune cells is clinically feasible, can produce deep remissions, and can be managed with toxicity profiles similar to *ex vivo* CAR-T approaches. Analogous vector-engineering strategies could be applied to myeloid-directed platforms carrying WT-*TP53* coding sequences.

## Recombinant adenoviral vectors modeled on rAd-p53

Adenoviral vectors provide a complementary DNA-based approach, with decades of oncology experience and a favorable safety profile when delivered regionally. Traditional Ad5 vectors have limited tropism for HSC due to low coxsackie and adenovirus receptor (CAR) expression. This limitation can potentially be overcome by fiber-knob modification (e.g., Ad5/35 chimeras), enabling CD34+ HSC targeting, or by adapter molecules that retarget the vector to alternative receptors such as CD46, CD123, or other myeloid antigens (48, 49). Ad.IL3, a CD123-targeting oncolytic adenovirus, has shown the ability to sustainably infect and suppress the proliferation of AML cells in preclinical models, underscoring the feasibility of leukemia-directed adenoviral therapy (50).

It is critical to distinguish replication-defective p53-replacement adenoviruses (such as rAd-p53/Gendicine^®^, in which the E1 replication cassette is deleted and replaced by a WT-*TP53* expression unit) from oncolytic adenoviruses (which retain replication competence and exert antitumor effects partly through tumor lysis) (11). The proposed “leukemia-adapted rAd-p53” would be a replication-defective vector relying entirely on receptor-mediated delivery of WT-*TP53* transgene. Evidence from oncolytic constructs such as Ad.IL3 demonstrates receptor-retargeting feasibility but does not directly establish efficacy or safety of replication-defective WT-*TP53* delivery in AML/MDS. These are distinct platforms requiring independent validation. A “leukemia-adapted rAd-p53” could thus comprise a replication-defective adenovirus encoding WT-*TP53* under a strong promoter, packaged in a capsid retargeted to CD123, CD33, or CD209. Upon binding and internalization into *TP53*-mutant leukemic cells, episomal *TP53* expression could restore p53 activity, potentially sufficient to drive apoptosis and enhance sensitivity to cytotoxic or alternative therapies. Tropism-modified adenoviral vectors have been explored in HSC editing and have shown efficient genome delivery without the integration risks associated with retroviral systems, further supporting their suitability as p53-replacement vehicles (49).

## Preclinical requirements for proof-of-concept and safety evaluation

Before clinical translation, several preclinical questions require rigorous evaluation in *TP53*-mutant models. AML PDX models engrafted with primary *TP53*-mutated blasts should be used to test CD33-, CD123-, or CD209-targeted WT-*TP53* vehicles. Effective transduction into the leukemic cells needs to be proven in adenoviral platforms. The optimal multiplicity of infection (MOI), defined as the proportion of agents to the target cells, should be examined, and dose calculation parameters should be defined (9). GFP reporters can be used in LNP mRNA platforms to measure effective mRNA delivery (46, 51). The duration of therapeutic effects should be measured, and endpoints, including leukemic burden, microenvironment remodeling, survival, and clonal dynamics, should be examined with and without sequential cytotoxic therapies.

It is essential to demonstrate that WT-*TP53* delivery can overcome the dominant-negative effects of *TP53* hotspot mutations, high VAF mutations, and multihit disease (52). Humanized mouse models with multilineage hematopoiesis will be required to systematically compare on-target efficacy versus off-tumor toxicity when targeting CD33, CD123, or CD209, including quantitative assessment of effects on leukemic cells and non-malignant myeloid compartments (38, 50).

To provide a more concrete experimental roadmap, the following sequential questions should be addressed: (1) Can CD33-, CD123-, or CD209-targeted LNPs or adenoviral vectors deliver WT-*TP53* constructs selectively to primary *TP53*-mutated AML blasts and leukemic stem/progenitor cells (LSCs), measured by GFP reporter or p53 protein expression? (2) Is WT-p53 protein expressed at levels sufficient to shift the WT:mutant stoichiometric ratio and activate canonical p53 target genes (e.g., CDKN1A/p21, PUMA, BAX)? (3) Does WT-*TP53* delivery result in measurable apoptosis, growth arrest, or chemosensitization across distinct *TP53* mutation classes (hotspot dominant-negative, null/truncating, monoallelic, biallelic)? (4) What is the IV biodistribution profile in humanized mice, and can miR-122 detargeting or surface shielding reduce hepatic off-target expression? (5) Does delivery to CD209+ DCs or macrophages cause measurable immunosuppression, and do transcriptional restriction strategies (e.g., CD33 promoter-driven *TP53*) effectively prevent off-target immune cell expression? These experiments will be necessary to establish the preclinical foundation required for IND-enabling studies.

## Outlook

The emerging convergence of p53 gene therapy, AML surface proteomics, and *in vivo* RNA/viral delivery to HSC creates a potential framework for direct p53 restoration in select *TP53*-mutated myeloid malignancies. rAd-p53 illustrates that p53 replacement is clinically feasible and can synergize with cytotoxic therapies. Surfaceome studies provide a growing catalogue of specific antigens that can be exploited to target leukemic myeloid stem cells. *In vivo* HSC gene-editing and CAR-T platforms demonstrate that systemic gene delivery to hematopoietic and immune compartments can be clinically achievable.

We emphasize that the strategy proposed here is explicitly speculative and extrapolatory. Each component – antigen-directed delivery, WT-*TP53* payload, and efficacy in *TP53*-mutated myeloid disease – draws on established prior work, but the integrated hypothesis has not been directly tested. Critical barriers including dominant-negative stoichiometry, liver sequestration, CD209-related immune toxicity, and the molecular heterogeneity of *TP53*-mutated AML/MDS must be systematically addressed in rigorous preclinical models before any clinical translation is considered. Within this landscape, CD33-, CD123-, and CD209-directed WT-*TP53* delivery – via optimized mRNA/LNP systems or tropism-modified adenoviral vectors – could represent a credible and testable strategy to address the unmet need of *TP53*-mutated myeloid malignancies.

## Data Availability

The original contributions presented in the study are included in the article/supplementary material. Further inquiries can be directed to the corresponding author.
